# Effect of low frequency magnetic fields on melanoma: tumor inhibition and immune modulation

**DOI:** 10.1186/1471-2407-13-582

**Published:** 2013-12-06

**Authors:** Yunzhong Nie, Leilei Du, Yongbin Mou, Zhenjun Xu, Leihua Weng, Youwei Du, Yanan Zhu, Yayi Hou, Tingting Wang

**Affiliations:** 1 Immunology and Reproduction Biology Lab, Medical School & State Key Laboratory of Pharmaceutical Biotechnology, Nanjing University, 210093 Nanjing, China; 2 Stomatological Hospital Affiliated Medical School, Nanjing University, 22 Hankou Road, 210093 Nanjing China; 3 National Laboratory of Solid Microstructures, Nanjing University, Nanjing, China; 4 Jiangsu Key Laboratory of Molecular Medicine, Nanjing University, Nanjing, China

**Keywords:** Magnetic fields, Melanoma, Immune function

## Abstract

**Background:**

We previously found that the low frequency magnetic fields (LF-MF) inhibited gastric and lung cancer cell growth. We suppose that exposure to LF-MF may modulate immune function so as to inhibit tumor. We here investigated whether LF-MF can inhibit the proliferation and metastasis of melanoma and influence immune function.

**Methods:**

The effect of MF on the proliferation, cell cycle and ultrastracture of B16-F10 in vitro was detected by cell counting Kit-8 assay, flow cytometry, and transmission electron microscopy. Lung metastasis mice were prepared by injection of 2 × 10^5^ B16-F10 melanoma cells into the tail vein in C57BL/6 mice. The mice were then exposed to an LF-MF (0.4 T, 7.5 Hz) for 43 days. Survival rate, tumor markers and the innate and adaptive immune parameters were measured.

**Results:**

The growth of B16-F10 cells was inhibited after exposure to the LF-MF. The inhibition was related to induction of cell cycle arrest and decomposition of chromatins. Moreover, the LF-MF prolonged the mouse survival rate and inhibited the proliferation of B16-F10 in melanoma metastasis mice model. Furthermore, the LF-MF modulated the immune response via regulation of immune cells and cytokine production. In addition, the number of Treg cells was decreased in mice with the LF-MF exposure, while the numbers of T cells as well as dendritic cells were significantly increased.

**Conclusion:**

LF-MF inhibited the growth and metastasis of melanoma cancer cells and improved immune function of tumor-bearing mice. This suggests that the inhibition may be attributed to modulation of LF-MF on immune function and LF-MF may be a potential therapy for treatment of melanoma.

## Background

The treatment of low frequency magnetic fields (LF-MF) is thought to be non-invasive and non-ionizing and even possess non-thermal effects on cells and tissues. Thus the possible effects of LF-MF on human health have been attractively explored. Interestingly, some studies showed that magnetic fields (MF) had a significant antitumor activity *in vitro* and *in vivo*. Sixty Hz sinusoidal MF could significantly inhibit cell growth and induce apoptosis in prostate cancer cells by cleaved caspase-3 and accumulated reactive oxygen species [1]. LF-MF (0.5-16.5 Hz) combined with a static MF could markedly suppress tumor growth in Ehrlich ascites carcinoma (EAC) model mice [2]. Recently, advanced hepatocellular carcinoma patients were treated with intrabuccally administered amplitude-modulated electromagnetic fields, and results showed that the treatment exhibited anti-tumor effects as well as safe and well tolerated [3].

Melanoma, derived from melanocytes, is one of the most highly invasive and metastatic tumors [4]. The incidence of malignant melanoma has increased dramatically in recent years [5]. Melanoma can spread "silently" at an early stage without any symptoms of metastasis, which become one of the major obstacles for achieving successful clinical chemotherapy, surgery and radiotherapy for the treatment of melanoma [6]. Therefore, novel therapeutic strategies are needed to overcome tumor metastasis and to improve the survival and prognosis of melanoma patients. Strikingly, it was reported that 50 Hz MF inhibited melanoma cell survival and reversed resistance to therapy through up-regulation of the anti-apoptotic protein BAG3 in melanoma cells [7]. The static MF (35 to 120 mT) also inhibited the growth of melanoma cells [8]. In addition, tumor growth in mice model was suppressed by a weak intensity MF (1-5 nT) [9]. It is thus obvious that specific frequency of MF indeed inhibits the growth of melanoma cancer.

Of note, immune system plays a crucial role in protecting the host against cancer. There is strong evidence for the existence of an effective cancer immunosurveillance process in human and mice. In tumor bearing host, however, immune system is often not able to execute effective responses, primarily because of negative regulatory mechanisms employed by growing cancer [10]. As is well known, regulatory T cell (Treg) is a specialized subset of CD4 T cells. In certain cancers the increased Treg numbers and/or function may promote cancer progression by interfering with immune surveillance. Increased Treg numbers correlated with worse prognosis in cancer patients [11]. The depletion of Treg led to a better anti-tumor immune response in the murine system [12]. Importantly, the role of MF on immune system had been reported. Phagocyte activity, ROS release and interleukin-1β (IL-1β) production were significantly promoted after continuous exposure to 50 Hz LF-MF (1mT) [13]. MF exposure negatively correlated with NK activity [14]. However, it is necessary to clarify modulation of MF on immune system.

We previously developed a revolving magnetic field system and found that the LF-MF (0.4 T, 7.5 Hz) inhibited gastric and lung cancer cell growth and altered midkine expression in cancer cells [15]. We suppose that exposure to MF may inhibit tumor growth through activation of immune system. In the present study, we thus investigated the effect of MF (0.4 T, 7.5 Hz) on B16-F10 melanoma cells *in vitro* and B16-F10 melanoma lung metastasis model *in vivo*. Furthermore, we examined the effect of MF on innate immune and adaptive immune system including immune cells number and cytokine profiles.

## Methods

### Experimental magnetic fields

**Figure 1:**
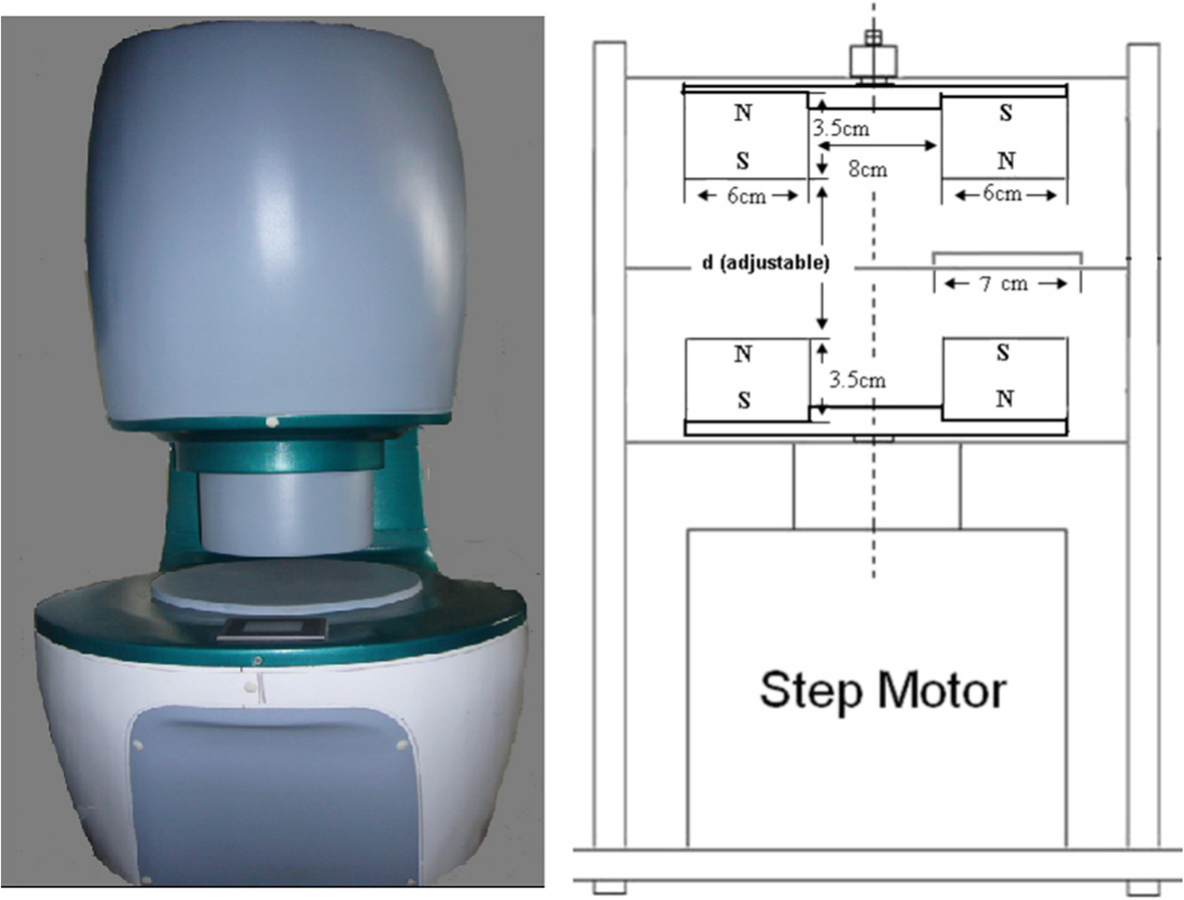
Magnetic field exposure system.

The construction of experimental magnetic fields has been described previously [15]. As shown in Figure 1, two pairs of fan-shaped NdFeB permanent magnets (N45, Innuovo, Dongyang, China) were embedded into a circular iron plate and arranged to establish MF. The bottom two magnets rotated at certain frequency driven by a step motor, which was controlled using a functional signal generator. The top two magnets rotated synchronously due to the strong magnetic interaction. Magnetic flux density was measured at the target site using a gauss meter (HT201, Hengtong, Shanghai, China). MF at the target site is alternative pulses with a maximum flux density of 0.4 T. The frequency of MF was 0-15 Hz (7.5 Hz was used in this study based on the previous experiments). This instrument was fabricated by the National Laboratory of Solid Microstructures, Nanjing University (Nanjing, China). Control cells and mice were placed in a similar apparatus except that there were two rotating iron plates instead of magnets (sham MF). For cell experiment, the entire magnetic apparatus was located in an environment with humidity and temperature controller.

### Cell culture

B16-F10 melanoma cells were obtained from the Shanghai Institute of Cell Biology (Shanghai, China). Cells were grown in RPMI-1640 medium (Gibco, Carlsbad, CA) with 10% fetal bovine serum (FBS) (Gibco, Carlsbad, CA) and 100 U/ml penicillin and 100 U/ml streptomycin (Amresco, Solon, OH, USA) at 37°C in a water-saturated atmosphere with 5% CO_2_.

### Cell apoptosis and cell cycle analysis

For cell apoptosis assay, cells were harvested, washed once with binding buffer (10 mM Hepes, 140 mM NaCl, 2.5 mM CaCl_2_) and stained with 5 μl Annexin V-FITC (eBioscience, San Diego, CA) for 15 min and 10 μl propidium iodide (PI, 20 μl g/ml) (eBioscience, San Diego, CA) for 10 min. For cell cycle analysis, cells were harvested, washed once with phosphate buffer saline (PBS), and fixed in 70% ethanol overnight. Staining for DNA content was performed with 50 mg/ml PI, 2%Triton-×100 and 1 mg/ml RNaseA for 30 min. Cells were analyzed by flow cytometry. Cell-cycle modeling was performed with Modfit 3.0 software (Verity Software House, Topsham, ME). Three independent experiments were carried out for cell apoptosis and cell cycle detection.

### CFSE labeling assay

Resuspend B16-F10 cells in pre-warmed PBS at a final concentration of 1 × × 1010^66^ / mL_○_ cells/ml Add 2 μL of 5 mM stock 5-(and -6) carboxyfluorescein diacetate succinimidyl ester (CFSE) (Invitrogen, Carlsbad, CA) solution per milliliter of cells for a final working concentration of 10 μM. Incubate the cells for 15 min at 37°C. Replace the loading solution with fresh, pre-warmed medium and incubate the cultures for another 30 min at 37°C. Wash the cells by resuspending the pellet in fresh media. Pellet and resuspend the cells in fresh media a further two times. For a total of three washes. After culture in a 5% CO_2_ incubator at 37°C for 5 days with sham MF or MF (2 h/day), the proliferation of B16-F10 cells was detected by flow cytometry.

### Cell counting Kit-8 assay

Cells (1 × 10^4^ cells per well) were incubated in 96-well culture plates (Corning Inc., New York, USA) in 100 μL of medium. After culturing for 5 days with sham MF or MF (2 h/day), 10 μL Cell Counting Kit-8 (CCK-8) (Dojindo Laboratories, Kumamoto, Japan) was added into each well. Absorbance was measured at 450 nm using a microplate reader.

### Transmission Electron Microscopy (TEM)

Exponentially growing B16-F10 cells were seeded into 10 cm dishes (Costar). After intermittent exposure to sham MF or MF for 5 days, cells were harvested, washed twice, fixed with 4% glutaraldehyde for 2 h, post-fixed with 1% OsO4, dehydrated in graded concentrations of ethanol, and then embedded in resin SPI-Pon 812 (Shell Chemical, Yuhuan, China). Ultra-thin sections were cut (80 nm), counterstained with lead citrate and uranylacetate, and then observed with a Philips CM 12 electron microscope (Philips, Eindhoven, Holland) at 80 kV. Percentage of positive cells in every 500 cells was counted.

### Quantitative real time polymerase chain reaction

Total RNA was isolated using Trizol reagent (Invitrogen, Carlsbad, CA) according to the manufacturer’s instructions. A total of 1 μg RNA was used as the template for single strand cDNA synthesis utilizing random primers and the Primescript reverse transcriptase (M-MLV, Takara, Japan). The cDNA was amplified using SYBR green PCR Mix (iTAP, Bio-Rad) on an ABI step-one plus sequence detection system (Applied Biosystems, Foster City, CA), programmed for 95°C for 10 min, then 40 cycles of 95°C for 15 s, 60°C for 30 s, and 72°C for 30 s. The amplification results were analyzed using StepOne Software (V2.1, Applied Biosystems) and the gene of interest was normalized to the corresponding β-actin results. The primer sequences are as following, Bcl2, (sense) 5'-CTCGTCGCTA CCGTCGTGACTTCG -3' and (anti-sense) 5'-AGATGCCGGTTCAGGTACTCA GTC-3'; Survivin, (sense) 5'-GTACCTCAAGAACTACCGCATC-3' and (anti-sense) 5'-GTCATCGGGTTCCCAGCCTTCC-3'; β-actin, (sense) 5'-GCGTGACATCAAAG AGAAGCT-3' and (anti-sense) 5'-ATGCCACAGGATTCCATACC-3'.

### Animals and experimental groups

4-6 week-old female C57BL/6 mice were purchased from the Animal Research Center of Yangzhou University, PR China. Mouse care and experimental procedures were performed under special pathogen-free conditions with standard rodent chow and water. The experiments were conducted according to institutional animal ethics guidelines. The protocol was approved by the Committee on the Ethics of Animal Experiments of Nanjing University Medical School affiliated Drum Tower Hospital ethics committee.

We induced lung metastases by injection of 2 × 10^5^ B16-F10 melanoma cells into the tail vein. Mice were divided into four groups, Sham MF Normal group (n = 6), the mice were normal and exposed to sham MF; MF Normal group (n = 6), the normal mice were exposed to MF; Sham MF Tumor mice group (n = 17), the mice were induced melanoma lung metastases and exposed to sham MF; and MF Tumor mice group (n = 17), the mice were induced melanoma lung metastases and exposed to MF. Three days after tumor challenged, mice were treated with sham MF or MF (0.4 T, 7.5 Hz, 2 h/day) for 43 days. The mice were sacrificed on days 46. Cardiac blood was collected from each mouse and centrifuged at 450 g for 20 min. Serum was obtained and stored at -70°C until use. The entire spleen, liver, lung, kidney and heart was removed from each mouse and measured. Splenic cells were collected from each mouse spleen for further experiment.

### Immunohistochemistry

Tumor tissues of tumor mice and lung tissues of normal mice were harvested, fixed in 10% buffered formalin, dehydrated, bisected, mounted in paraffin, and sectioned for immunohistochemistry (IHC). Immunostaining was performed on 6-μm tissue sections using strept-avidin-biotin staining kit (Boster). For antigen retrieval, slides were heated by microwave in 0.01 mol/L Tri-sodium citrate buffer. Nonspecific binding sites were blocked with 5% BSA for 30 min and endogenous peroxidase activity was suppressed by treatment with 3% H_2_O_2_ in methanol for 30 min. Sections were exposed to anti-rabbit polyclonal Ki-67 (1:100, Abcam, Cambridge, MA, USA) overnight at 4°C. 3,3-diamino-enzidine was used as chromogen (Boster). Counterstaining was done with hematoxylin. Negative control sections were incubated with PBS instead of anti-Ki-67 antibodies. In each step, samples were washed with PBS. Cells in five different microscope fields of each Ki-67 staining tissue were used to calculate positive cells.

### Cytokines analysis

Serum cytokines were analyzed by the Proteome Profiler Array ARY006 (R&D systems, Minneapolis, MN, USA) according to manufacturer’s instructions. Briefly, 1 ml mixed serum was obtained from five mice of each group. Serum diluted in 0.5 ml array buffer was mixed with a 15 ml cocktail of biotinylated detection antibodies. The serum/antibody mixture is then incubated with the mix buffer. Any cytokine/detection antibody complex present is bound by its cognate immobilized capture antibody on the membrane. Following a wash to remove unbound material, Streptavidin-Horseradish Peroxidase and chemiluminescent detection reagents were added. Array images are from 5 min exposures to X-ray film. The array allows to detect 40 kinds of proteins: CXCL13, C5a, G-CSF, GM-CSF, CCL1, CCL11, sICAM-1, IFN-γ, IL-1α, IL-1β, IL-1ra, IL-2, IL-3, IL-4, IL-5, IL-6, IL-7, IL-10, IL-13, IL-12p70, IL-16, IL-17, IL-23, IL-27, IP-10, CXCL11, KC, M-CSF, CCL2, CCL12, CXCL9, CCL3, CCL4, CXCL2, CCL5, CXCL12, CCL17, TIMP-1, TNF-α, and TREM-1. Integral optical density (IOD) was analyzed by Gel-Pro Analyzer 4 (Toyobo, Osaka, Japan) software. The IOD of interest cytokines were normalized to the corresponding positive control IOD results.

### Immune cells detection

We For the collection of splenic cells, single-cell suspension were dissociated by gently pressing the organ through a fine, 50 μm-nylon mesh, and cells were collected by centrifugation at 300 g for 5 min. Erythrocytes were removed by treating the splenic cells with red blood cell lysis buffer (0.15 M NH_4_Cl, 1.0 mM KHCO_3_, 0.1 mM Ethylene Diamine Tetraacetie Acid (EDTA), pH 7.2) for 5 min and washing twice with cold PBS. 1 × 10^6^ cell were suspended in PBS and incubated with related anti-mouse antibodies for 30 min at 4°C, then washed twice with fluorescence activating cell sorter (FACS) washing buffer. Data were acquired on FACS Vantage SE (FACSCalibur, Becton Dickinson, San Jose, CA) and analyzed with CellQuest software (CellQuest Pro, Becton Dickinson). The following antibodies used for flow cytometry: anti-CD3-PE-Cy5.5, anti-CD4-FITC, anti-CD8-PE, anti-CD11c-PE, anti-CD40-APC and anti-isotype-specific control Abs were purchased from eBioscience (eBioscience, San Diego, CA). Three independent cells were carried out for detection of each immune cell.

### Statistical evaluation

Values were expressed as mean ± S.E.M. Statistical analysis was performed using Mann-Whitney U test to compare the mean values between two groups. In case of survival curve, the data were analyzed by the log-rank test. Values of *P* < 0.05 were considered to be statistically significant. Statistical analysis was done using the SPSS 11.5 software program (SPSS, Chicago, IL).

## Results

### LF-MF inhibits the proliferation of B16-F10 cells

**Figure 2:**
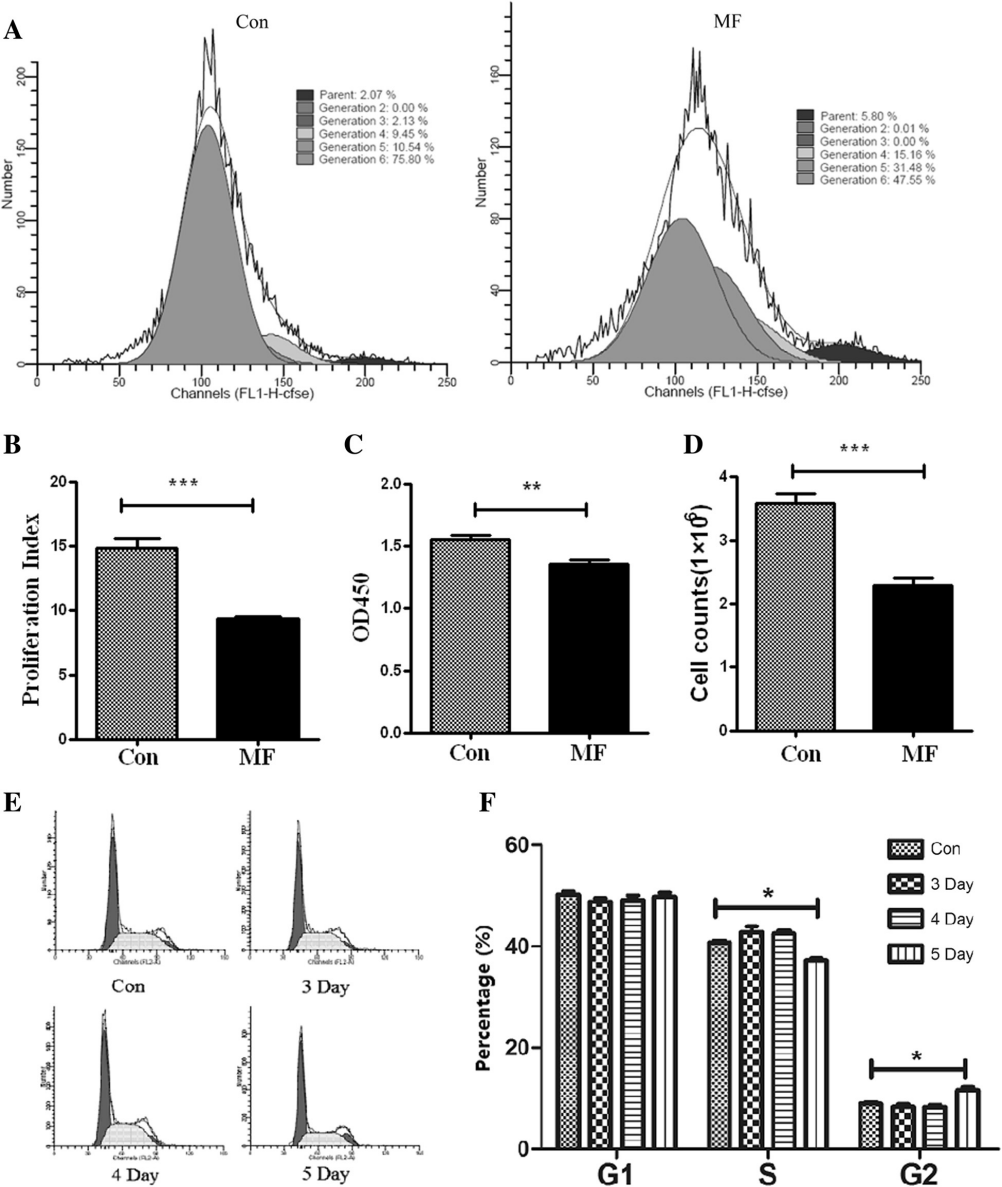
**LF-MF inhibits the proliferation and induce cell cycle arrest in B16-F10 cells. (A)** CFSE proliferation assay analyzes the proliferation of B16-F10 after exposed to sham LF-MF (Con) or LF-MF (2 h/day, 5 days); The means of proliferation index using CFSE assay **(B)**; CCK-8 assay **(C)** and simple cell counting **(D)** detects the cell proliferation in sham LF-MF (Con) and LF-MF (2 h/day, 5 days). **(E)** Flow cytometric analysis of cell cycle after exposure to LF-MF for 3, 4 and 5 days. **(F)** The mean percentage of G1, S and G2 phase cells. All the experiments were repeated three times. Data are expressed as means ± S.E.M.

Firstly, we analyzed the effect of LF-MF (0.4 T, 7.5Hz) on the proliferation of B16-F10 cells using CFSE labeling assays. As shown in Figure 2A and B, after 5 days exposure, the proliferation of B16-F10 (proliferation index = 9.35) was suppressed by LF-MF compared to sham LF-MF (proliferation index =14.87). Then, the inhibitory effect of the MF was confirmed by CCK8 assay and simple cell counting, and the results showed that LF-MF slightly but significantly inhibited the proliferation of B16-F10 cells (Figure 2C).

### LF-MF promotes B16-F10 apoptosis and arranges the process of cell cycle arrest in vitro

The effect of LF-MF on the process of cell cycle was also examined. After 3 and 4 days exposure, no difference in the G0/G1, S, and G2/M was observed between sham LF-MF group and LF-MF group. While prolong this exposure to 5 days, the S-phase rate was significantly decreased from 40.76% to 37.24% and the G2/M-phase rate was significantly increased from 8.9% to 11.6% (Figure 2E and F). The apoptosis cells were slightly increased from 15.98% to 21.36% after 5 days exposure to LF-MF (Additional file 1: Figure S1A and B). Then we checked the expression of genes associated with apoptosis, Bcl2 and survivin, and found that mRNA expressions of the genes were not changed after exposure to LF-MF (Additional file 1: Figure S1C and 1D).

### LF-MF alters the ultrastructure of B16-F10 cells

**Figure 3:**
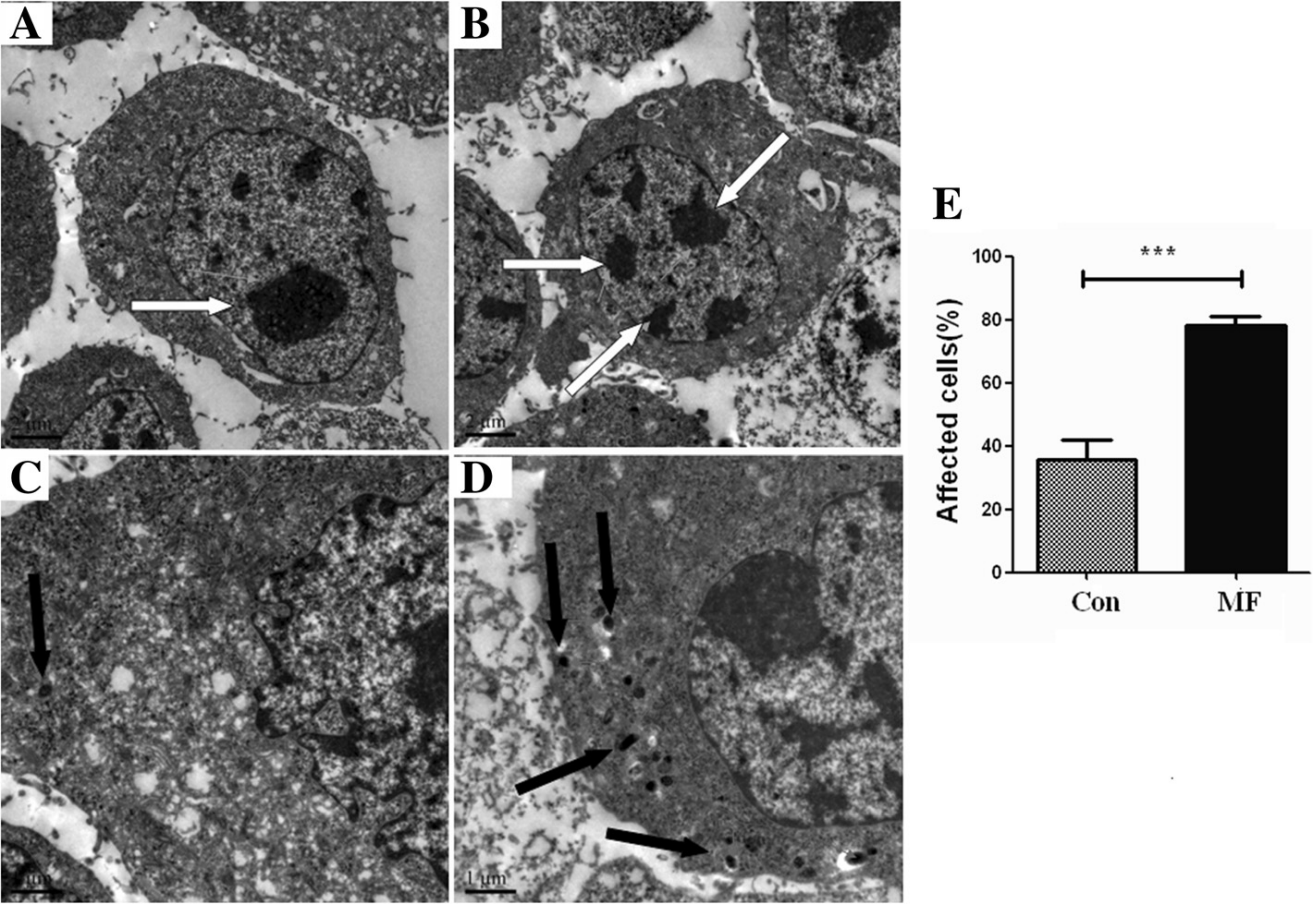
**LF-MF alters the utrastructure of B16-F10 cells.** Cell ultrastructure of B16-F10 cells exposure to Sham LF-MF, original magnification **(A)** 500×; **(C)** 1000×; Cell ultrastructure of B16-F10 cells exposure to LF-MF, **(B)** 500×; **(D)** 1000×. Cells were observed by transmission electron microscopy. Percentage of positive cells in every 500 cells were counted **(E)**. Compared with control cells, the treated cells were characterized by the breaking down of chromatin (white arrow) and black granule accumulation (black arrow).

The ultrastractural alteration of B16-F10 cells was observed by transmission electron microscopy (TEM) after exposure to MF for 5 days. Compared with the sham exposed cells, the LF-MF-treated cells were characterized by chromatin decomposed (white arrow), and the decomposed chromatins reached to the boundary of the karyotheca (Figure 3A and B). Also we found that the black granules, which represent the senescence degree of cells, were accumulated in cytoplasm (black arrow) in LF-MF-treated cells (Figure 3C and D).

### LF-MF raises survival rate and inhibit tumor proliferation in mice model

**Figure 4:**
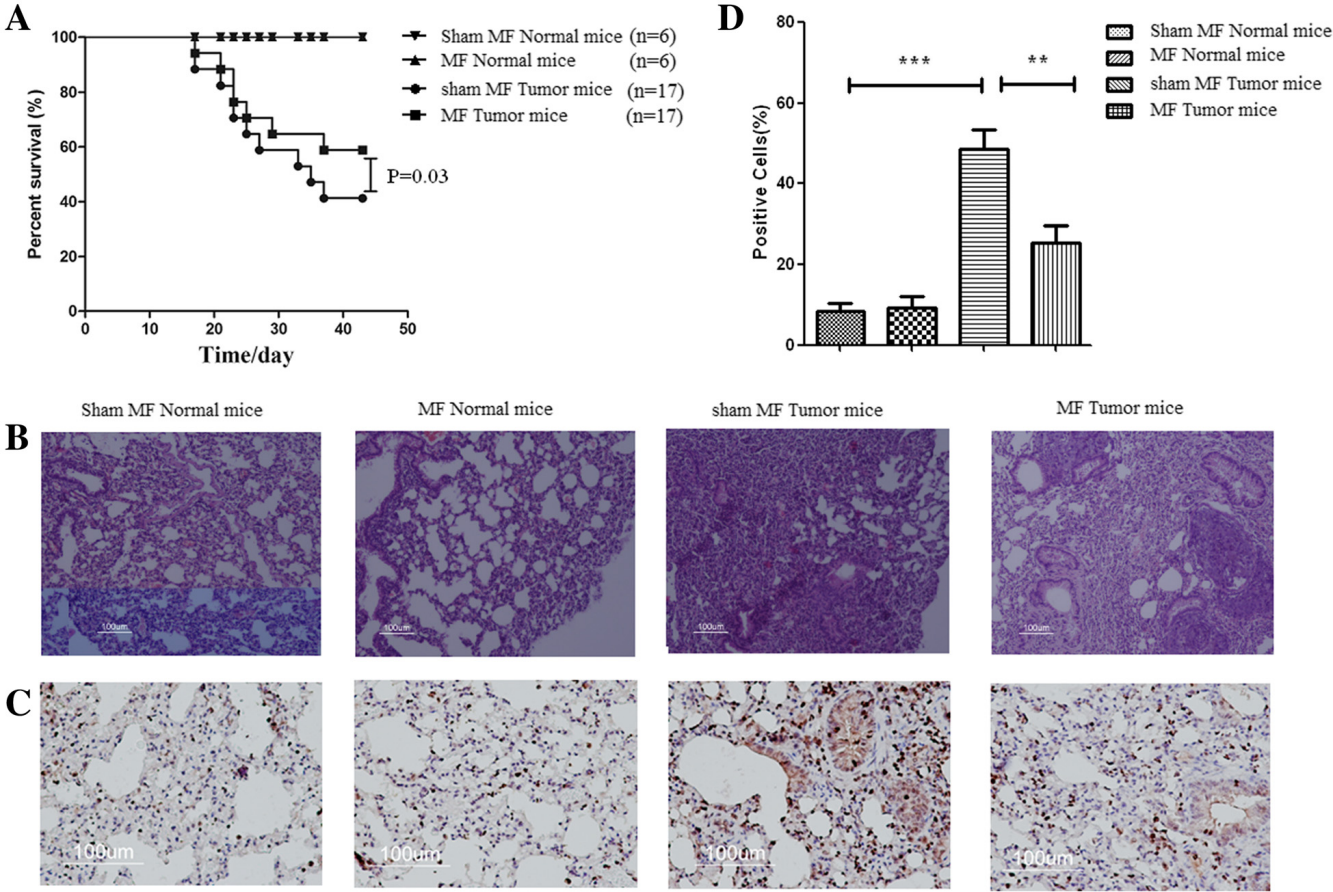
**LF-MF raises survival rate and inhibit tumor proliferation *in vivo*. (A)** Statistical analysis of survival after intravenous challenge of C57BL/6 mice with B16-F10 cells, and treated with LF-MF. Mice were treated byLF-MF for 43 days, 2 h/day; **(B)** HE of mice lung in different groups. **(C)** Immunohistochemistry analyzes the expression of Ki-67 in tumors of tumor-bearing mice and in lungs of normal mice. **(D)** Proportion of Ki-67 positive cells in different groups. Five different microscope fields of each group were examined for Figure 4B and C, and cells in five different microscope fields of each Ki-67 staining tissue were used to calculate positive cells in Figure 4D.

To investigate whether the LF-MF influences the growth of B16-F10 cells *in vivo*, we constructed the lung metastasis of melanoma mice model. Normal mice and tumor-bearing mice were exposed to sham LF-MF and LF-MF (0.4 T, 7.5Hz), respectively. After exposure to LF-MF for 43 days (2 h/day), the sham tumor-bearing mice group experienced 58.8% mortality, while LF-MF tumor-bearing mice group only showed 41.2% mortality, and the normal mice showed no mortality after sham LF-MF or LF-MF treatment (Figure 4A). HE and immunohistochemistry analysis of lung showed that the numbers of Ki-67 positive cells were significantly higher in tumor-bearing mice groups than normal mice groups, whereas LF-MF tumor-bearing mice group showed a lower number of Ki-67 positive cells, compared with the sham LF-MF tumor-bearing mice group (Figure 4B and C).

Next, we detected the alteration of the organs in mice. After challenged with B16-F10 cells, a significant tumefaction was showed in spleen and kidney, while this abnormal tumefaction could be reversed after exposure to LF-MF (Additional file 2: Figure S2A, B and E). The masses of lung, liver, and heart were mildly altered in both normal mice and tumor-bearing mice after LF-MF exposure (Additional file 2: Figure S2C, D and F).

### LF-MF regulates the production of inflammatory cytokines in mice serum

**Figure 5:**
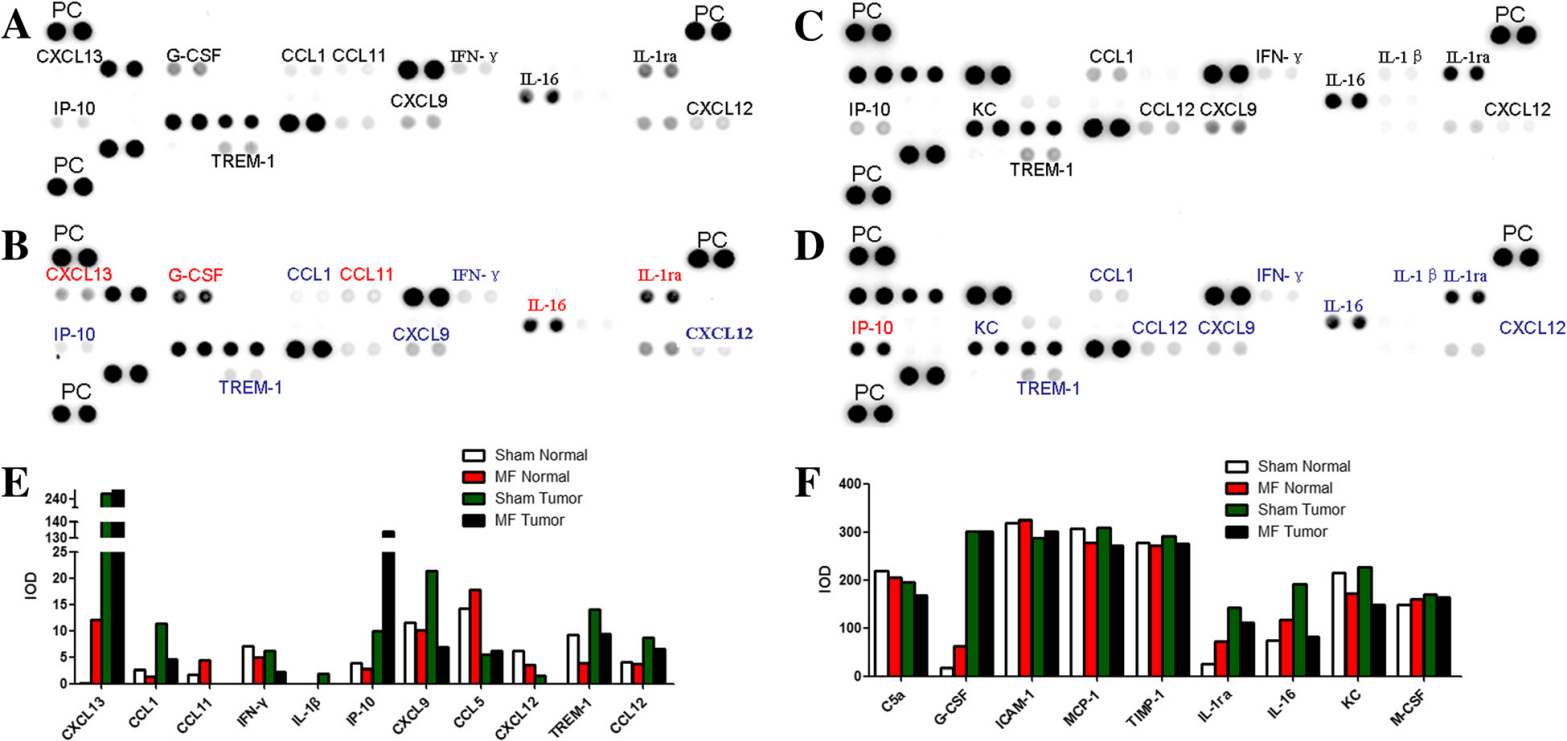
**LF-MF regulates the production of inflammatory cytokines in mice serum.** Using 1 ml mixed serum of each group, cytokine profiles from sham normal group **(A)**, LF-MF Normal group **(B)**, sham tumor group **(C)** and MF tumor group **(D)** were detected by cytokine array, respectively. The high-intensity spots in the three corners are positive controls (PC). In **A** and **B**, selected cytokines are labeled: red, cytokines that increased compared with sham normal group; blue, cytokines that decreased compared with sham normal group; In **C** and **D**, selected cytokines are labeled: red, cytokines that increased compared with sham normal group; blue, cytokines that decreased compared with sham normal group. **(E)** and **(F)** show the mean IOD for each group.

Since LF-MF inhibited the growth of B16-F10, we next attempted to clarify the effect of the LF-MF on immune response. Inflammatory cytokines in mice serum were measured by Proteome Profiler Array. After mice challenged with B16-F10, the cytokines production was totally changed. Compared with the sham LF-MF normal mice group, amount of some cytokines was increased in sham LF-MF tumor-bearing mice, including CXC chemokine ligand (CXCL)13, CC chemokine ligand (CCL) 1, interleukin (IL)-1β, IFN-γ-inducible protein (IP)-10, CXCL9, triggering receptor expressed on myeloid cells (TREM)-1, CCL12, granulocyte colony-stimulating factor (G-CSF), IL-1rα and IL-16, while only few cytokines were decreased, including CCL11, CCL5 and CXCL12 (Figure 5A, C, E and F). The LF-MF also influenced production of cytokines. In normal mice groups, CXCL13, CCL11, CCL5, G-CSF, IL-1ra and IL-16 were up-regulated and CCL1, IFN-γ, CXCL12 and KC were down-regulated after LF-MF exposure (Figure 5A, B, E and F). Whereas in tumor-bearing mice groups, most of cytokines were decreased after LF-MF exposure, including KC, CCL1, IFN-γ, CXCL9, CXCL12, TREM-1, CCL12, IL-1rα and IL-16. And the only increased cytokines was IP-10 (Figure 5C, D, E and F).

### LF-MF promotes T cells polarization in spleen

To detect whether the immune cells were regulated after exposure to LF-MF, the proportions of T lymphocytes in spleen cells was analyzed by flow cytometry. In normal mice groups, there were no differences in the proportions of CD3+, CD3 + CD4+ and CD3 + CD8+ T cells between sham exposed and exposed groups. After challenged with B16-F10 in mice, the proportions of T cells in spleen were significantly decreased from 49.15% to 18.64% for CD3+ T cells (Additional file 3: Figure S3A), from 26.35% to 8.99% for CD3 + CD4+ T cells (Additional file 3: Figure S3B), and from 17.15% to 5.88% for CD3 + CD8+ T cells (Figure 5C). After LF-MF exposure, the proportions of CD3+, CD3 + CD4+ and CD3 + CD8+ T cells in tumor-bearing mice were increased to 24.0%, 13.28% and 7.46%, respectively (Additional file 3: Figure S3A, B and C).

### LF-MF prevents Treg cell differentiation in spleen

**Figure 6:**
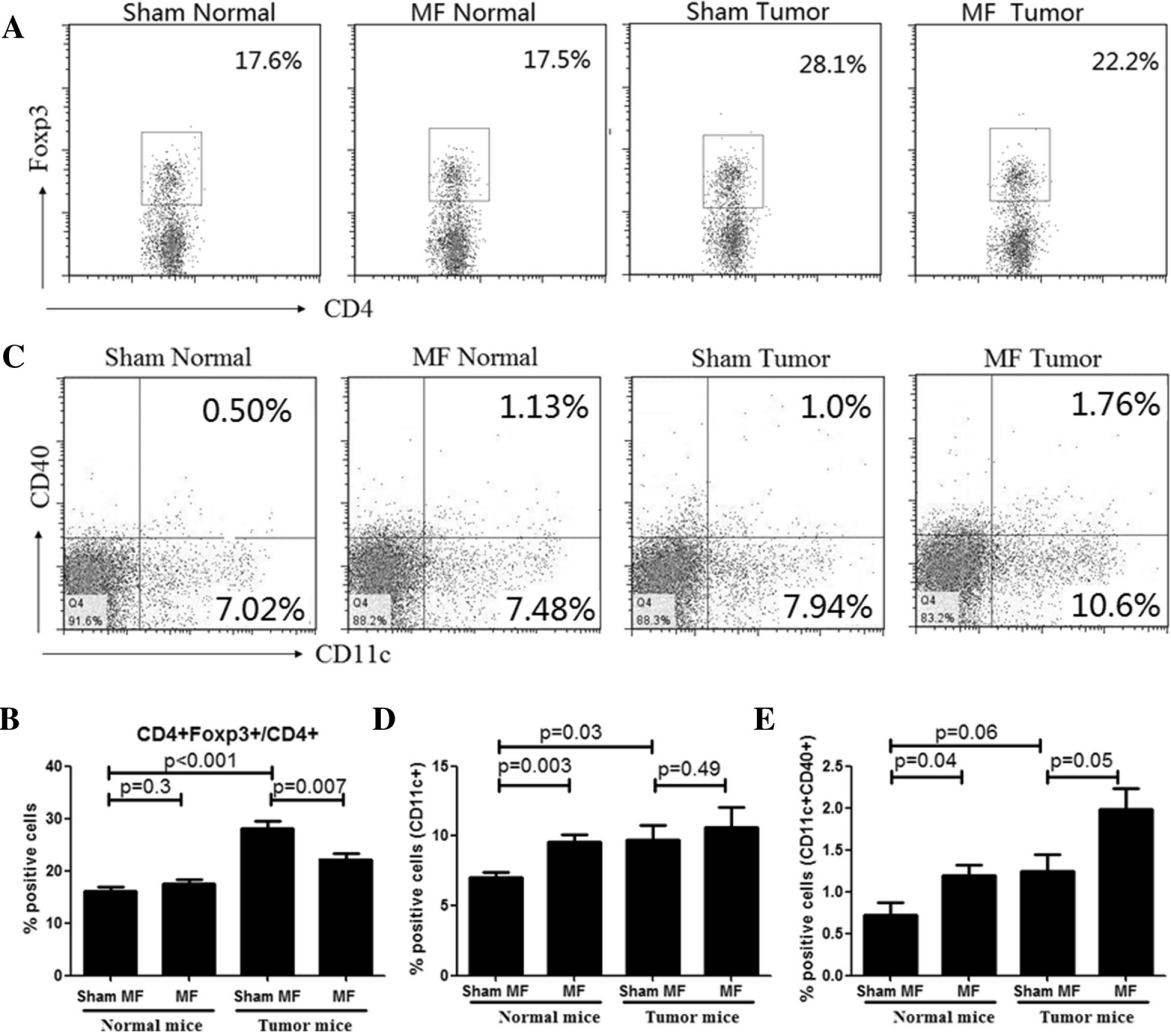
**LF-MF prevents Treg cell differentiation and enhance the expression of CD40 in dendritic cells in spleen. (A)** Flow cytometry analyzes the population of Treg cells in total CD4+ cells in splenetic lymphocytes; **(B)** the mean percentage of CD4 + Foxp3+ T lymphocytes in CD4+ T cells for each group; **(C)** Flow cytometry analyzes CD11c + CD40+ dendritic Cells (DC) proportion in splenetic lymphocytes for each group. **(D)** The mean proportion of CD11c + DCs in splenetic lymphocytes for each group. **(E)** The mean proportion of CD11c + CD40+ DCs in splenetic lymphocytes for each group. All the experiments were repeated three times. Data are expressed as means ± S.E.M.

After exposure to LF-MF, CD3 + CD4+ T cells significantly increased in tumor-bearing mice. Treg is known as a pro-tumor T cell [16]. Then the proportion of Treg cells in spleen was analyzed by flow cytometry. As shown in Figure 6A and B, the proportion of Treg cells in sham normal mice spleen was 16.15% of all CD4+ T cells, while sham tumor-bearing mice exhibited a 1.74-fold increase of Treg cells (28.1% of the CD4+ population). After exposed to LF-MF, the proportion of Treg cells in normal mice (17.5% of the CD4+ population) had not increase compared with sham normal mice, while the proportion of Treg cells in tumor-bearing mice was declined to 22.2%, which was significantly less than that in sham MF tumor mice.

### LF-MF enhances the expression of CD40 in dendritic cells

We further tested the alteration of dendritic cells (DC) after exposure to LF-MF. We found that the percentage of DC in spleen, characterized by CD11c + expression, was significantly increased in LF-MF normal mice (9.57% of spleen lymphocytes) and sham LF-MF tumor-bearing mice (9.75% of spleen lymphocytes), compared with the sham normal mice (7.03% of spleen lymphocytes). In LF-MF tumor-bearing mice, the percentage of DC was closed to the sham tumor-bearing mice, about 10.63% of spleen lymphocytes (Figure 6C and D). Moreover, we also examined the expression of CD40 on DC. After exposure to LF-MF, the percentage of CD40+ DC was significantly increased in both normal mice and tumor-bearing mice, from 0.72% to 1.19%, and from 1.25% to 1.98%, respectively (Figure 6C and E).

## Discussion

Treatment of melanoma is a major challenge due to the limited number of therapeutic options available. In the present study, we explored the tumor inhibitory effect of LF-MF (0.4 T, 7.5Hz) in melanoma B16-F10 cell line and melanoma lung metastasis, and found that the LF-MF inhibited the proliferation in B16-F10 cells and induced a G2/M-phase arrest. The LF-MF also influenced the ultrastructure of B16-F10 cells. Furthermore, we found that survival rate of B16-F10-challenged mice was improved after LF-MF exposure, and the tumor proliferation in lung was also inhibited. In addition, the function of innate immune cells and adaptive immune cells was significantly modulated after LF-MF exposure.

LF-MF (0.4 T, 7.5 Hz) inhibited the growth of cancer cells, which is consistent with our previous study [15]. It was reported that MF inhibited the cell growth by promoting apoptosis and arranging the cell cycle [17-19]. Tt is thus clear that the inhibitory effect of MF was significant but not specific in melanoma. Moreover, the LF-MF also exerted the inhibition with a delay in progression of cells from S to G2/M phase of the cell cycle. The apoptosis cells were slightly increased after 5 days exposure to LF-MF. However, the expression of genes associated apoptosis (Bcl2 and Survivin) showed no difference between sham LF-MF and LF-MF group. This result suggests that the inhibitory effect of LF-MF may not mainly be attributed to the induction of apoptosis. As is well known, both apoptosis and autophagy may also play an important role in the inhibitory effect of LF-MF. Thus, it will be needed to explore other supporting experiments such as BrdU labeling and TUNEL in further study. In addition, B16-F10 cell exhibited significant ultrastructural changes after exposure to LF-MF for 5 days, including the parted cell chromatin and the accumulation of black granules. Previous studies reported that static magnetic fields affected cell ultrastructure [20]. These results imply the cells may undergo lysis and apoptosis and the aging of B16-F10 may be improved after this LF-MF exposure.

The survival rate of melanoma model mice was elevated after LF-MF exposure, which is in agreement with the previous studies [2]. It was reported that the lung metastases were the primary characteristics after intravenous challenge of C57BL/6 mice with melanoma cells [21]. Furthermore, we detected broadly expression of Ki-67 the lungs of tumor mice by immunohistochemistry analysis and found the expression of Ki-67 was significantly decreased after LF-MF exposure. It is known that Ki-67 protein is a cellular marker for proliferation and strictly associated with cell proliferation [22]. In addition, the abnormal tumefaction of spleen and kidney in tumor challenging mice were modulated after LF-MF exposure. Spleen tumefaction is a familiar phenomenon in tumor challenging mice. The modulative effect of LF-MF on spleen in B16-F10 challenging mice suggests that the LF-MF may reverse the effect of intravenous B16-F10 cells. These results are in agreement with previous studies that static and LF-MF-induced inhibition of human breast adenocarcinoma, but not embryonal lung fibroblast [23].

As the most important surveillance in organism, immune system has three primary roles in the prevention of tumors: protect the host from virus-induced tumors, prevent the establishment of an inflammatory environment conducive to tumorigenesis and specifically identify and eliminate tumor cells [24]. On the other hand, immune surveillance is not always successful, tumors can escape immune surveillance and ‘edit’ immune system to block antitumor adaptive and innate responses and promote tumor progression. It is believed that Treg cells have the potent ability to suppress host immune responses and tumor cells can recruit Treg cells to inhibit anti-tumor immunity in the tumor microenvironment, thus helping the tumor escape from the immune surveillance [16]. In this study, we found that the frequency of T cells and DCs in mice was significantly increased after LF-MF exposure, while the number of Treg cells was decreased. Furthermore, the LF-MF abolished the benefit factors for tumor growth and slowed the growth of the tumor. The anti-tumor cytokine, IP-10 [25], was significantly accumulated, while the pro-tumor cytokines, KC [26], CCL1 [27], CCL12 [28] and CXCL12 [29] were decreased after LF-MF exposure. In addition, we also found that T cells in the spleen were significantly changed in mice with tumor, but LF-MF affected the proportion of different T cells in spleen. this suggests that spleen, as an important immune organ, may be involved in anti-tumor role during LF-MF treatment. All these results support our hypothesis that the LF-MF may modulate the immune response and inhibit the growth of cancer cells. However, the biological function of T cell alteration during LF-MF treatment will be needed to clarify in the following study.

## Conclusions

In conclusion, our present study demonstrated that the LF-MF (0.4 T, 7.5 Hz) inhibited the growth of B16-F10 in vitro. Furthermore, the LF-MF elevated the survival rate, and inhibited the proliferation of B16-F10 cells in lung metastasis model mice. The inhibition in vitro may be attributed to the suppression of proliferation, increase of G2/M phase, slightly induction of apoptosis and decomposition of the chromatins. The LF-MF also modulated the immune response including the levels of cytokine production and functions of innate immune cells and adaptive immune cells. The elevation of survival rate in tumor-bearing mice may be related to the improvement of immune function. Taken together, our study proved that LF-MF inhibits the growth of melanoma and may provide a potential therapy for treatment of melanoma.

## Competing interests

The authors declare that they have no competing interests.

## Authors’ contributions

Conceived and designed the experiments: YH, TW, YN. Performed the experiments: YN, LD, YM, LW, ZX. Analyzed the data: YN, TW, YZ. Contributed reagents/materials/analysis tools: YD. Wrote the manuscript: YN, YH, TW. All authors read and approved the final manuscript.

## Pre-publication history

The pre-publication history for this paper can be accessed here:

http://www.biomedcentral.com/1471-2407/13/582/prepub

## Supplementary Material

Additional file 1: Figure S1 LF-MF influences cell apoptosis and expression of apoptotic associate genes in B16-F10. (A) and (B) Flow cytometric analysis of apoptosis in B16-F10 cells after exposure to LF-MF for 3, 4 and 5 days. Relative exprssion of Bcl2 (C) and Survivin (D) in B16-F10 after exposure to LF-MF. Data are expressed as means ± S.E.M. Click here for file

Additional file 2: Figure S2 LF-MF modulates the masses of the mice organs. (A) Images showing spleen enlarge after intravenous challenge of C57BL/6 mice with B16-F10 cells and treatment with LF-MF for 43 days. The mean weights of spleen for each group are depicted to the right of the image. (C), (D), (E) and (F) show the mean weights of lung, liver, kidney and heart for each group. (B) HE analysis of spleen in different groups. Data are expressed as means ± S.E.M. Click here for file

Additional file 3: Figure S3 LF-MF promotes T lymphocytes polarization in spleen. Flow cytometry analyzes CD3+ (A, left), CD3 + CD4+ (B, left) and CD3 + CD8+ (C, left) T lymphocytes in splenetic lymphocytes for each group. The mean percentage of CD3+, CD3 + CD4+ and CD3 + CD8+ T lymphocytes in splenetic lymphocytes for each group are depicted to the right of the images. Three independent experiments were carried out. Data are expressed as means ± S.E.M. Click here for file
