# Supplementary figures and images for: Effect of low frequency magnetic fields on melanoma: tumor inhibition and immune modulation
Source: BMC Cancer. 2013 Dec 6;13:582. doi: 10.1186/1471-2407-13-582 (PMC4029221; doi:10.1186/1471-2407-13-582)

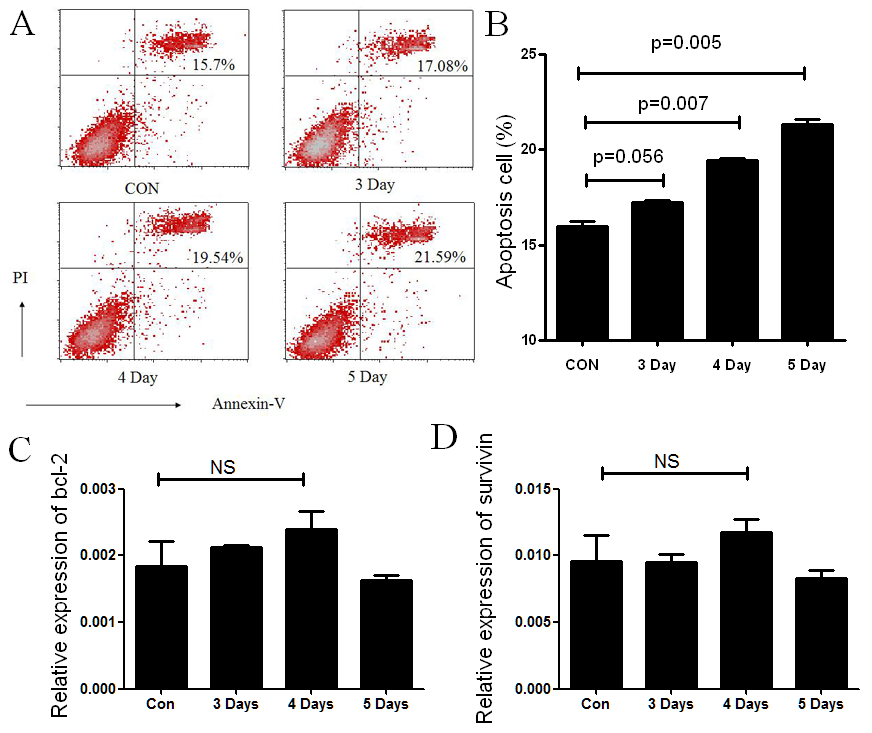

Supplement: Additional file 1: Figure S1 — LF-MF influences cell apoptosis and expression of apoptotic associate genes in B16-F10. (A) and (B) Flow cytometric analysis of apoptosis in B16-F10 cells after exposure to LF-MF for 3, 4 and 5 days. Relative exprssion of Bcl2 (C) and Survivin (D) in B16-F10 after exposure to LF-MF. Data are expressed as means ± S.E.M. [file 1471-2407-13-582-S1.tiff]

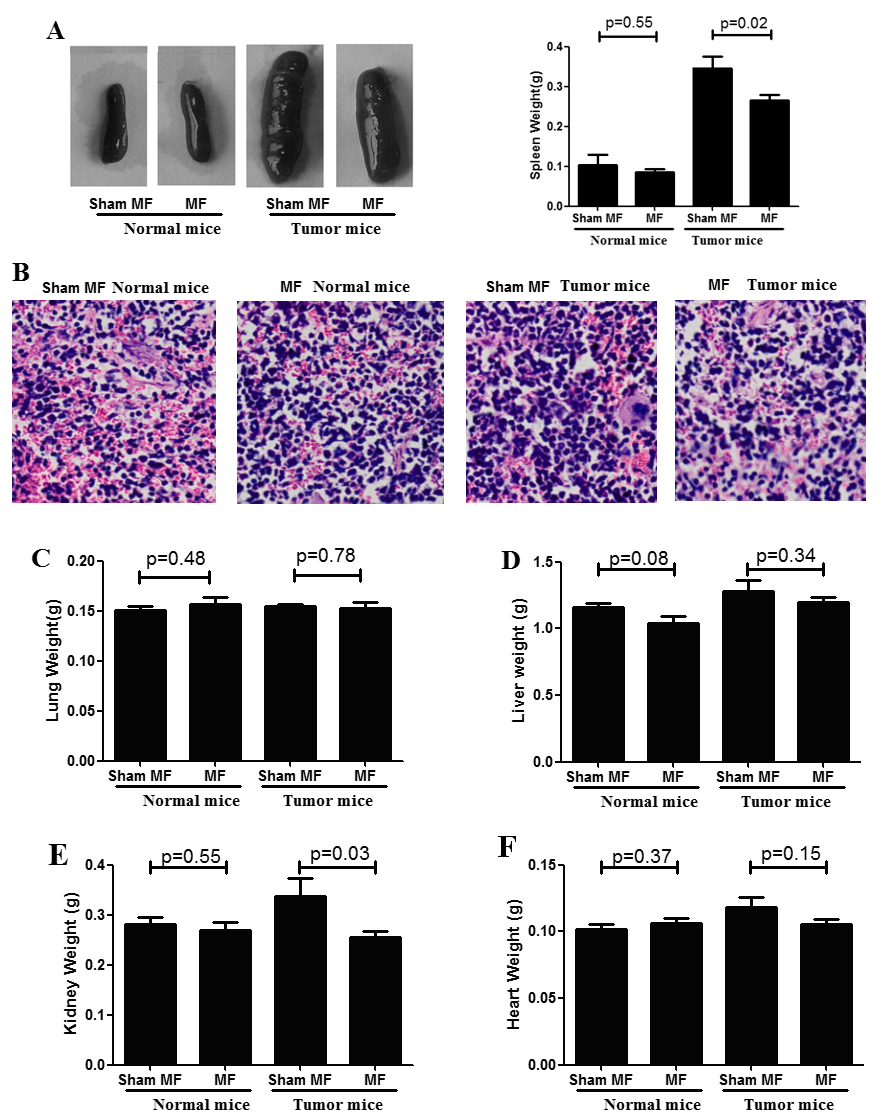

Supplement: Additional file 2: Figure S2 — LF-MF modulates the masses of the mice organs. (A) Images showing spleen enlarge after intravenous challenge of C57BL/6 mice with B16-F10 cells and treatment with LF-MF for 43 days. The mean weights of spleen for each group are depicted to the right of the image. (C), (D), (E) and (F) show the mean weights of lung, liver, kidney and heart for each group. (B) HE analysis of spleen in different groups. Data are expressed as means ± S.E.M. [file 1471-2407-13-582-S2.tiff]

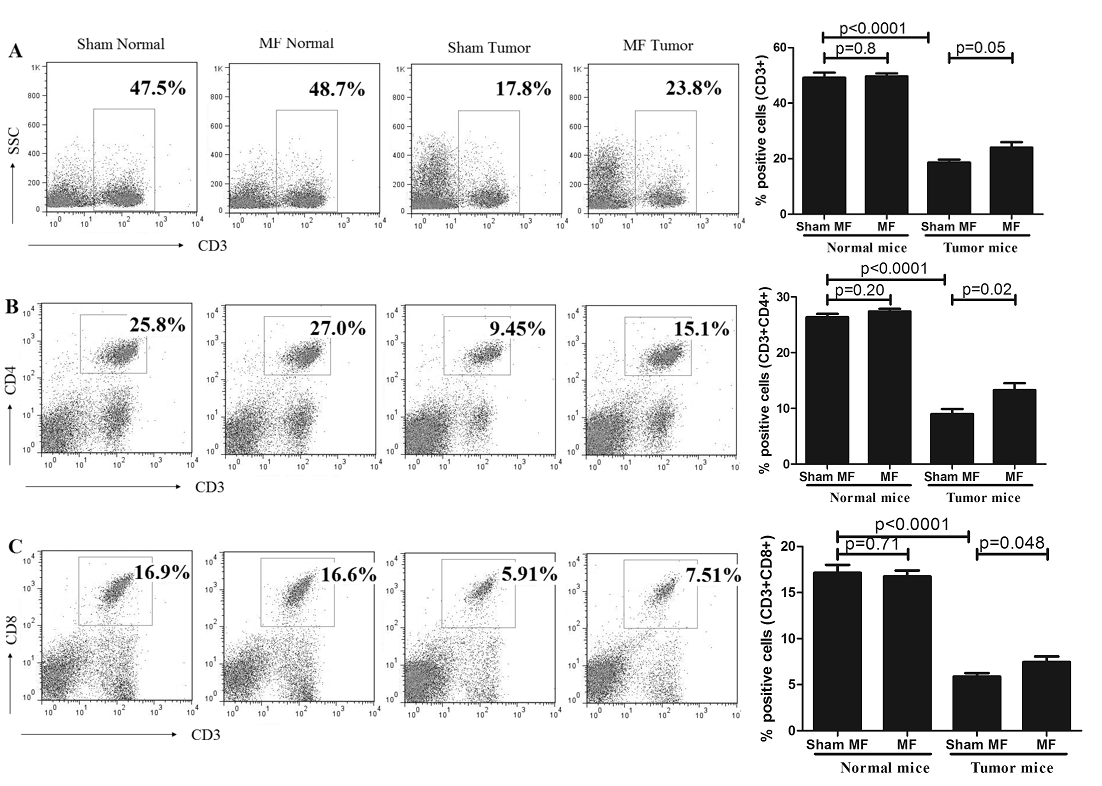

Supplement: Additional file 3: Figure S3 — LF-MF promotes T lymphocytes polarization in spleen. Flow cytometry analyzes CD3+ (A, left), CD3 + CD4+ (B, left) and CD3 + CD8+ (C, left) T lymphocytes in splenetic lymphocytes for each group. The mean percentage of CD3+, CD3 + CD4+ and CD3 + CD8+ T lymphocytes in splenetic lymphocytes for each group are depicted to the right of the images. Three independent experiments were carried out. Data are expressed as means ± S.E.M. [file 1471-2407-13-582-S3.tiff]
